# Synaptic GABA release prevents GABA transporter type-1 reversal during excessive network activity

**DOI:** 10.1038/ncomms7597

**Published:** 2015-03-23

**Authors:** Leonid Savtchenko, Maria Megalogeni, Dmitri A. Rusakov, Matthew C. Walker, Ivan Pavlov

**Affiliations:** 1 UCL Institute of Neurology, Queen Square, London WC1N3BG, UK

## Abstract

GABA transporters control extracellular GABA, which regulates the key aspects of neuronal and network behaviour. A prevailing view is that modest neuronal depolarization results in GABA transporter type-1 (GAT-1) reversal causing non-vesicular GABA release into the extracellular space during intense network activity. This has important implications for GABA uptake-targeting therapies. Here we combined a realistic kinetic model of GAT-1 with experimental measurements of tonic GABA_A_ receptor currents in *ex vivo* hippocampal slices to examine GAT-1 operation under varying network conditions. Our simulations predict that synaptic GABA release during network activity robustly prevents GAT-1 reversal. We test this in the 0 Mg^2+^ model of epileptiform discharges using slices from healthy and chronically epileptic rats and find that epileptiform activity is associated with increased synaptic GABA release and is not accompanied by GAT-1 reversal. We conclude that sustained efflux of GABA through GAT-1 is unlikely to occur during physiological or pathological network activity.

## Abstract (web summary)

Membrane depolarization during increased neuronal activity as seen during epilepsy has been suggested to easily reverse neuronal GABA transporters. Here the authors use modelling and experimental data and challenge this view by showing that synaptic GABA release during excessive neuronal firing averts reversal of GABA uptake.

## Graphical abstract

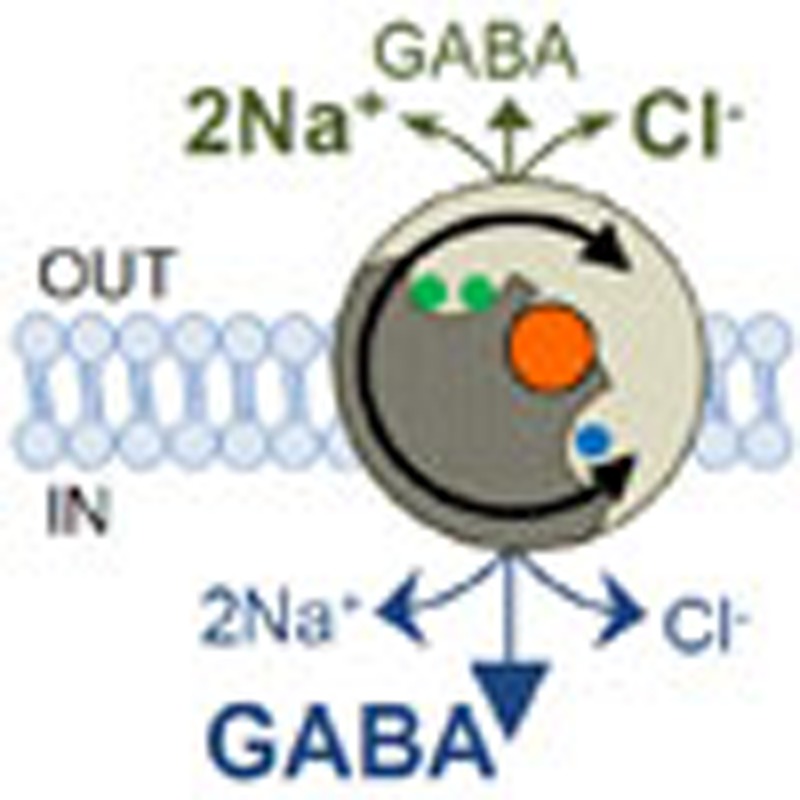

Gamma-aminobutyric acid (GABA) release and uptake hold the key to the excitation–inhibition balance in the brain. In addition to fast synaptic inhibition, GABA can mediate a slower form of signalling through the persistent activation of slowly desensitizing, high-affinity extrasynaptic GABA_A_ receptors (GABA_A_Rs)^1^,^2^. This type of tonic GABA_A_R-mediated conductance can be detected in the majority of hippocampal and cortical neurons, in which it controls cell excitability (for example, ref. 3). Importantly, the magnitude of tonic conductance varies with local network activity^4^,^5^, depending on the kinetic equilibrium between GABA release and uptake. The resultant fluctuations in extracellular GABA can, in turn, regulate neuronal excitability and the synchronization of neuronal networks^6^. Activity of GABA transporters therefore plays a key role in regulating neuronal and network activity.

Four different subtypes of GABA transporters (GATs) have been identified: GAT-1, 2, 3 and betaine GABA transporter (corresponding in mice to GAT-1, 3, 4 and GAT-2, respectively). The most abundantly expressed transporter in cortical structures is GAT-1, which is primarily responsible for neuronal GABA uptake. The other highly expressed cortical GABA transporter is the predominantly glial GAT-3 (refs 7, 8). Although transporter molecules normally clear GABA from the extracellular space, they can under certain conditions operate in the reverse mode^9^,^10^,^11^,^12^,^13^. GAT-1 and GAT-3 are similar in their stoichiometry: each GABA molecule (a zwitterion at physiological pH) is co-translocated with two Na^+^ ions and one Cl^−^ ion, thus making GABA transport electrogenic. Therefore, the transmembrane concentration gradients of Na^+^, Cl^−^ and GABA, together with the cell membrane potential, determine the direction of GABA transport. How the efflux of GABA through reversal of its transporters, in particular of neuronal GAT-1, contributes to the extracellular GABA concentrations remains controversial.

Although there have been no direct measurements of the reversal potential of GATs (*E*_GAT_) in brain tissue, indirect experimental evidence and theoretical estimations suggest that under baseline conditions transporters are near equilibrium, reviewed in Richerson and Wu^11^. This is often used to argue that GABA transporter reversal will occur with only moderate cell depolarization and during trains of action potentials^14^,^15^. It has been suggested that transporter-mediated GABA release can thus provide compensatory feedback that suppresses neuronal excitability through enhanced tonic inhibition during epileptiform discharges^16^. However, during intense network activity, there will also be an increased synaptic release of GABA and consequently an increase in the extracellular GABA concentration, which should stimulate GATs to transport GABA into cells. Therefore, there remains significant uncertainty concerning the mode of action of GATs during increased network activity.

*In vivo*, the inhibition of GABA transport during K^+^-induced neuronal depolarization produces a larger increase in ambient GABA concentration than does depolarization alone^17^. This observation questions whether the efflux of GABA through transporters indeed occurs during excessive neuronal firing (although astroglial release of GABA through GAT-3 has been suggested^18^,^19^,^20^). Understanding the roles of neuronal GABA transport in the face of increased network activity has important implications for therapeutic targeting of neuronal GATs. For example, if neuronal transporters indeed operate in the reverse mode during epileptiform discharges, the use of the antiepileptic drug tiagabine, which inhibits GAT-1, could have a paradoxical pro-epileptic effect by decreasing seizure-related rises in GABA.

To address these issues, here we determine the impact of network and neuronal activity both on GAT-1 operation and on the extracellular GABA concentration. We explore the established kinetic model of GAT-1 to argue, first, that the transporter should remain largely inactive at low ambient GABA concentrations and, second, that the synaptic release of GABA during intense network activity should robustly prevent GAT-1 from operating in the reverse mode. We then demonstrate in acute slice experiments that epileptiform discharges generated in the hippocampus of either healthy or chronically epileptic rats markedly increase synaptic GABA release thus preventing GAT-1 reversal. Together these findings imply that hippocampal GAT-1 transports GABA into neurons even in the face of increased network activity and widespread neuronal depolarization.

## Results

### GAT-1 activity is sensitive to ambient GABA concentration

**Figure 1:**
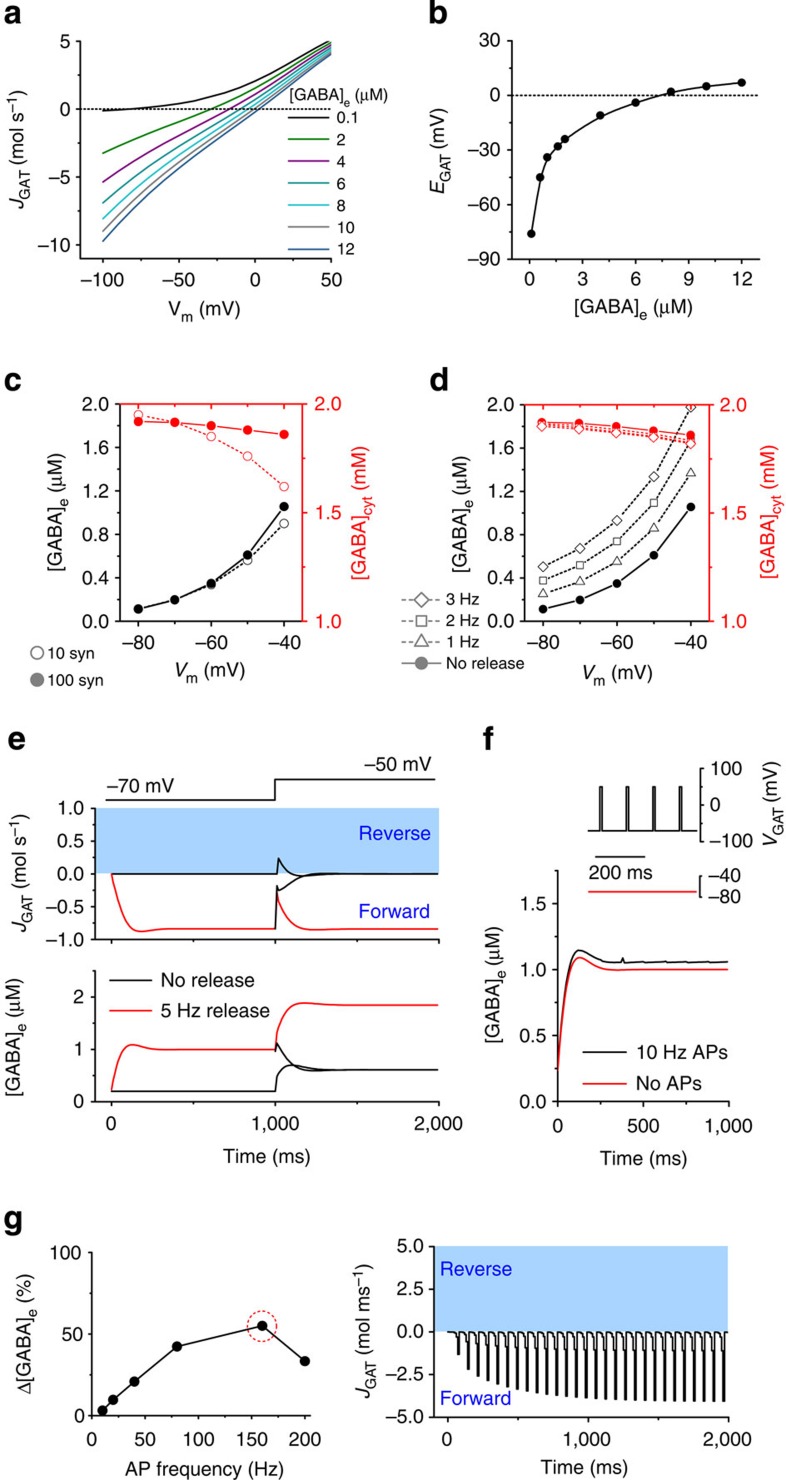
GAT-1 activity and [GABA]_e_ simulations. (**a**) Current–voltage relationship of the steady-state GAT-1-mediated current (*J*_GAT_, molecules s^−1^; negative values—forward mode, positive values—reverse mode) at different concentrations of extracellular GABA (in μM). (**b**) Dependence of GAT-1 reversal potential (*E*_GAT_) on the concentration of extracellular GABA. (**c**) Membrane potential dependence of the steady-state cytosolic ([GABA]_cyt_; red symbols) and extracellular GABA concentrations ([GABA]_e_; black symbols) in the absence of synaptic release. Filled circles—for 100 synapses, open circles—for 10 synapses. (**d**) Membrane potential dependence of the steady-state [GABA]_cyt_ and [GABA]_e_ at various rates of synaptic release. (**e**) The effect of depolarization on the dynamics of GAT-1 operation and [GABA]_e_ with (red) and without (black) synaptic GABA release. (**f**) The effect of presynaptic depolarization due to action potentials on the accumulation of extracellular GABA. Inset: simulated voltage changes. (**g**) Left: an increase in [GABA]_e_ due to presynaptic spike-induced depolarization at different firing frequencies (% change compared with [GABA]_e_ in the absence of simulated presynaptic action potentials). Right: GAT-1 current dynamics at 160 Hz presynaptic firing frequency.

To examine the impact of membrane depolarization and extracellular GABA concentrations ([GABA]_e_) on transporter operation in steady state, we adapted a previously established kinetic model of GAT-1 (ref. 21; Fig. 1a). Two important features emerged. First, at the low [GABA]_e_ observed experimentally under baseline conditions both *in vivo* and *in vitro*^22^, the current–voltage relationship of the transporter displayed marked outward rectification. Thus, there is little transporter activity when the membrane potential (*V*_m_) is below the spiking threshold. Second, *E*_GAT_ is highly sensitive to changes in ambient GABA at low [GABA]_e_ (Fig. 1b). Therefore, even a small increase in [GABA]_e_ readily pushes *E*_GAT_ to suprathreshold *V*_m_, suggesting that GAT would be operating in the forward mode on detection of GABA release by even depolarized neurons. Furthermore, [GABA]_e_ rises reduce the rectification, thus further facilitating GABA uptake (Fig. 1a).

### Activity-dependent GABA release prevents GAT-1 reversal

To determine how GAT-1 operation relates to the presence of synaptic GABA release, we adapted the above GAT-1 kinetic model for simulations of GABA concentration dynamics in the extracellular space and synaptic terminals. In the model, we placed 100 equally spaced GABAergic synapses each attached to a 22-μm-long axon segment in a 1,000-μm^3^ section of simulated hippocampal tissue, in accord with previous estimates^23^. Each synapse had ~1,200 GAT molecules and axonal segments had a linear density of 640 GATs  μm^−1^ (ref. 24) (see Methods). Since the overall GABA content in the brain remains relatively constant, we did not consider GABA breakdown and synthesis mechanisms in our model. We also assumed negligible impact of GABA diffusion from neuronal soma on the average steady-state GABA levels in synaptic terminals at the timescale of simulated events.

As expected, in agreement with previous findings in hippocampal neurons^15^,^16^,^25^, our simulations indicate that, in the absence of synaptic GABA release, membrane depolarization should increase the steady-state ambient GABA concentration due to transporter-mediated efflux (Fig. 1c). These [GABA]_e_ increases may be limited by the amount of cytosolic GABA to which the transporters are exposed. With the given overall GABA content available inside the synaptic terminals, both the cytosolic and the external GABA concentrations will be determined by the ratio of the tissue volume fractions occupied by the extracellular space and by the synaptic boutons. With an estimated extracellular volume fraction of ~0.14 (refs 26, 27) and realistic synaptic environment used in our simulations (see Methods for details), the depletion of intracellular GABA seems unlikely to prevent depolarization-induced [GABA]_e_ rises. However, decreasing the synapse density (and therefore the total amount of GABA that can be distributed between synaptic boutons and the extracellular space) reduced the effect of cell depolarization on [GABA]_e_ increases. The lower synapse density was also accompanied by a more pronounced depletion of the cytosolic GABA pool (Fig. 1c).

Simulations have shown that when synaptic GABA release is intact, [GABA]_e_ increases at all membrane potentials. This increase is more pronounced for faster rates of release. Similar to the above simulations, for any given rate of release, [GABA]_e_ is higher at more depolarized *V*_m_ (Fig. 1d). Expectedly, increasing or decreasing the density of GAT-1 (ref. 28) shifted [GABA]_e_ downwards or upwards, respectively (Supplementary Fig. 1).

Our model explores two potential sources for extracellular GABA, synaptic release and GAT-1 transporter-mediated efflux. We therefore set out to determine the relationship between the dynamics of GATs operation and [GABA]_e_ either with or without synaptic GABA release (Fig. 1e). As expected, in the absence of GABA release, transporters remain inactive. Neuronal membrane depolarization under these conditions leads to a brief reversal of GABA transport. Transporter-mediated efflux, however, ceases once a new steady-state GABA level is achieved. Sustained synaptic release, on the contrary, leads to uninterrupted GABA uptake (and a higher baseline [GABA]_e_) regardless of *V*_m_.

### GABA uptake during abnormal network activity

What if GABA release is abruptly terminated while cells become depolarized, which might potentially happen after or during a seizure (when some interneurons may enter a depolarization block or transiently exhaust their release machinery)? The model predicts that these conditions do not lead to GAT-1 reversal, but rather to decreased uptake and, eventually, to a complete cessation of GABA uptake as GABA drops to its non-release steady-state level. When synaptic GABA release persists during depolarization, [GABA]_e_ simply equilibrates to a new level after a transient decrease in transporter inward current, and transporters continue to operate in the forward mode (Fig. 1e).

Another common pathophysiological scenario is when large increases in neuronal activity are accompanied by intracellular Cl^−^ accumulation^29^,^30^ and/or action potential-associated Na^+^ influx^31^. Still, even in the case of a substantial and sustained increase of internal Cl^−^ concentration ([Cl^−^]_in_) and internal Na^+^ concentration ([Na^+^]_in_) the model predicts that GAT-1 is unlikely to reverse in the face of synaptic release (Supplementary Fig. 2a, Methods).

One important consequence of higher internal Cl^−^ and Na^+^ is that the efficiency of the transporter operation (in terms of transmembrane fluxes) in the forward mode becomes profoundly reduced (Supplementary Fig. 2b). Also, as neurons become loaded with Cl^−^ and Na^+^, the transporters will not be able to maintain the extracellular GABA concentration in the nM range at physiologically relevant cell membrane potentials (that is, above −80 mV, white area in Supplementary Fig. 2c). Furthermore, because an intracellular rise in Cl^−^ and Na^+^ causes the increase in the steady state [GABA]_e_, cytosolic GABA depletion on depolarization appears to be more pronounced (Supplementary Fig. 2d). This, however, has no bearing on the prevalence of direct transporter mode.

Since the majority of neuronal GAT-1 molecules are located on the axons and synaptic terminals of interneurons^7^, their operation may also be affected by large transient membrane depolarizations accompanying each round of action potential-dependent synaptic GABA release. To evaluate the impact of such a mechanism, we compared [GABA]_e_ attained when transporters experienced 2-ms-long voltage spikes (120 mV in amplitude) approximating action potentials, with that in the absence of spikes (Fig. 1f). The release rate in each synapse was set to half that of the rate of action potentials, to mimic a release probability of 0.5 observed in hippocampal interneurons^32^. Varying firing frequencies up to 200 Hz (the rate achieved by fast spiking interneurons), we found that [GABA]_e_ was increased by the presence of presynaptic action potentials; and the maximal change (55% increase) in [GABA]_e_ was at 160 Hz (Fig. 1g). This suggests that voltage-dependent changes in the efficiency of GABA transport may considerably alter [GABA]_e_ at high rates of release. However, even the maximal effect of action potential-dependent depolarization on extracellular GABA concentration did not trigger reversed GAT-1 transport (Fig. 1g).

Our simulations, therefore, suggest that synaptic GABA release results in GAT-1 operating in forward mode even during neuronal depolarization and rapid repetitive presynaptic firing. The prediction is therefore that during extremes of network activity, such as epileptiform activity, the transporters should not reverse. We tested this experimentally in the acute hippocampal slice preparation.

### GABA sensed by pyramidal neurons depends on GAT-1 activity

**Figure 2:**
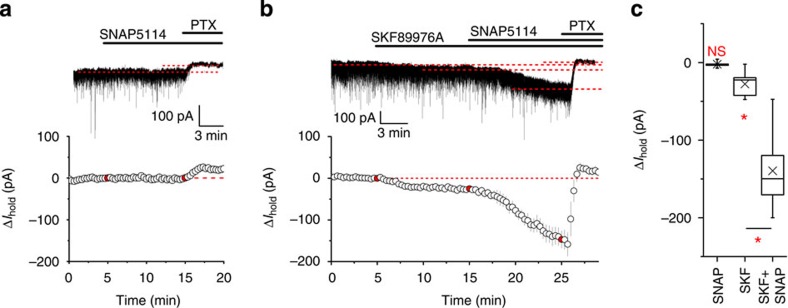
Tonic GABA_A_R currents in the presence of GAT inhibitors. (**a**) Top: a typical trace showing the effect of GAT-3 inhibitor SNAP5114 (100 μM) on the holding current (*I*_hold_) of a hippocampal CA1 pyramidal neuron. Bottom: averaged time course of five experiments. Data points in red indicate drug application times; PTX, picrotoxin. (**b**) Top: a typical experiment in which tonic GABA_A_R-mediated currents were induced by consequent application of GAT-1 (SKF89976A; 30 μM) and GAT-3 (SNAP5114; 100 μM) inhibitors. Bottom: averaged time course of six experiments. Data points in red indicate drug application times. (**c**) Box plot (boxes, 25–75%; whiskers, minimum–maximum, lines, median; × , mean) showing changes in *I*_hold_ caused by application of GAT inhibitors (SNAP5114: *n*=5, *P*=0.3, compared with baseline, paired *t*-test; SKF89976A: *n*=11, *P*=0.0002, compared with baseline, paired *t*-test; SKF89976A+SNAP5114: *n*=6, *P*=0.0014, compared with SKF89976A alone, paired *t*-test). Note high [Cl^−^]_in_ internal solution in these experiments (*V*_hold_=−70 mV); NS, not significant; *, significant changes; error bars on time course plots, s.e.m.

The relative contribution of the main cortical GABA transporters GAT-1 (neuronal) and GAT-3 (glial) to GABA uptake varies depending on brain region and cell type^5^,^17^,^33^,^34^. We first tested their impact on [GABA]_e_ in the hippocampal area CA1 using GAT inhibitors (Fig. 2). Whole-cell patch-clamp recordings were performed from pyramidal neurons in the presence of CGP55845 (1 μM), NBQX (20 μM), APV (50 μM) and tetrodotoxin (1 μM) to block GABA_B_ and ionotropic glutamate receptors, as well as action potential-dependent GABA release. Neurons were held at −70 mV and recorded using high Cl^−^ internal solution (see Methods). Application of the GAT-1 inhibitor SKF89976A (30 μM) produced a significant (Holm–Bonferroni correction) inward shift of holding current (Δ*I*_hold_; −27.7±4.7 pA; *n*=11, *P*=0.0002, paired *t*-test; Fig. 2b,c). No apparent significant (Holm–Bonferroni correction) changes in *I*_hold_ were observed on application of the GAT-3 inhibitor SNAP5114 (100 μM; −2.2±1.9 pA; *n*=5, *P*=0.3, paired *t*-test; Fig. 2a,c), confirming that under baseline conditions, with GAT-1 operational, GAT-3 has little effect on the GABA concentration detected by principal neurons in the hippocampus^17^,^19^. The same concentration of SNAP5114 applied following inhibition of the GAT-1 transporter resulted in a rapid significant (Holm–Bonferroni correction) increase in the inward current, indicating that GAT-3-mediated GABA uptake regulates [GABA]_e_ around pyramidal cells in the absence of GAT-1 activity (Δ*I*_hold_: −113.7±17.8 pA; *n*=6, *P*=0.0014, paired *t*-test compared with SKF89976A alone; Fig. 2b,c).

### Epileptiform bursts are associated with massive GABA release

**Figure 3:**
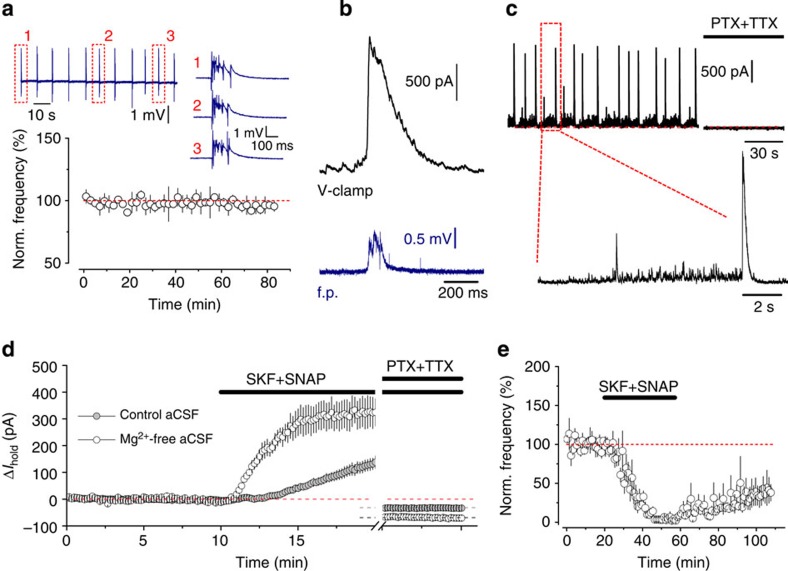
Synaptic GABA release during epileptiform activity. (**a**) Regular stereotypic epileptiform discharges (average frequency: 0.15±0.05 Hz; *n*=3) induced in Mg^2+^-free aCSF. Insert: sample field potential recording traces. (**b**) An example of a large GABA_A_R-mediated current in a CA1 pyramidal neuron (black, whole-cell voltage clamp, *V*_hold_=0 mV) associated with a field potential (f.p.) burst (blue). (**c**) Typical recording of GABA_A_R-mediated drive onto a pyramidal neuron (*V*_hold_=0 mV) during epileptiform activity. PTX, picrotoxin; TTX, tetrodotoxin. (**d**) Time course of *I*_hold_ changes induced by co-application of SKF89976A and SNAP5114 in control and Mg^2+^-free aCSF (rate of *I*_hold_ increase calculated 1–5 min after GAT inhibitors application in 0 Mg^2+^: 1.1±0.2 pA s^−1^; in control: 0.16±0.04 pA s^−1^; *n*=4 for each condition; *P*=0.004, *t*-test). (**e**) GAT inhibitors suppress the frequency of epileptiform discharges (3.6±2.6% of baseline; *n*=4, *P*<0.001, paired *t*-test). Error bars, s.e.m.

To address whether GABA transport reverses during extremes of network activity, we induced regular recurrent epileptiform discharges by using Mg^2+^-free artificial cerebrospinal fluid (aCSF)^35^. Under these conditions, the majority of slices developed stereotypical spontaneous interictal-like field potential bursts that persisted at a stable frequency for at least 1 h (Fig. 3a). To monitor the GABA_A_R-mediated currents in neurons during on-going epileptiform activity, pyramidal cells were voltage clamped at 0 mV, close to the reversal potential of glutamate receptor-mediated currents, using an intracellular solution containing 8 mM Cl^−^ (calculated Cl^−^ reversal potential, *E*_Cl_=−72 mV). Simultaneous field potential and whole-cell recordings revealed that each field potential burst was accompanied by a concomitant GABA_A_R-mediated transient (Fig. 3b,c). These GABA_A_R-mediated currents in pyramidal cells reached up to 1.5–2 nA in amplitude, while, between bursts, spontaneously occurring inhibitory post-synaptic currents (IPSCs) were two orders of magnitude smaller. We also noted that field potential recordings underestimate the duration of underlying burst discharges as these large, interictal-like event-associated GABAergic currents were considerably longer than bursts detected by extracellular electrodes, and often burst-associated large IPSCs were heralded by a flurry of smaller amplitude IPSCs at high frequencies (Fig. 3c).

Intense GABA release associated with epileptiform activity was also reflected in the rapid accumulation of extracellular GABA on inhibition of GAT-1 and GAT-3. The rate of increase of the tonic GABA_A_R-mediated current was significantly faster in ‘epileptic’ slices than in quiescent ones (*n*=4 for each condition, *P*=0.004; *t*-test, Fig. 3d). Blocking action potential-mediated neurotransmitter release and GABA_A_Rs, with tetrodotoxin and picrotoxin respectively, at the end of each experiment revealed larger tonic GABA_A_R currents in slices in which epileptiform activity was generated (*P*=0.016, *t*-test). Co-application of both GAT inhibitors almost completely abolished epileptiform discharges. This effect was partly reversible on drug washout (Fig. 3e).

Therefore, we conclude that even brief recurrent epileptiform population discharges in the hippocampus are accompanied by prolonged activation of interneurons leading to a marked increase in GABA release.

### GAT-1 inhibition does not reduce tonic GABA_A_R conductance

**Figure 4:**
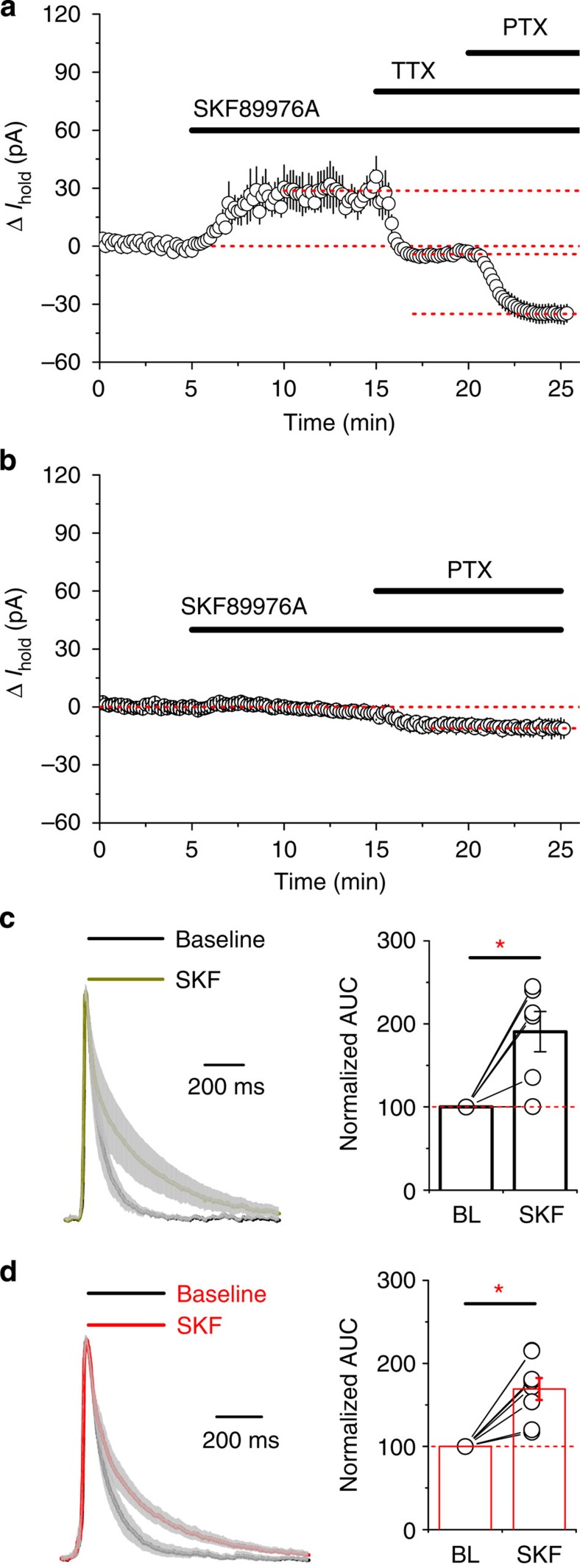
GAT-1 does not reverse during epileptiform activity. (**a**) Changes in tonic GABA_A_R-mediated currents in CA1 pyramidal neurons following application of SKF899976A during ongoing epileptiform activity (*n*=6; PTX, picrotoxin; TTX, tetrodotoxin; error bars, s.e.m.). (**b**) The effect of GAT-1 inhibition on tonic GABA_A_R-mediated currents in CA1 pyramidal neurons in the absence of synaptic GABA release (slices preincubated in 1 μM concanamycin; *n*=6; error bars, s.e.m.). (**c**,**d**) Mean normalized traces of GABA_A_R transients (grey: s.e.m.) show that SKF89976A similarly prolongs burst-associated GABA_A_R transients in pyramidal neurons from control (**c**; area under the curve, AUC, increases from 88.8±18.2 to 188.6±53.9 ms; *n*=6; *P*=0.038, paired *t*-test) and epileptic hippocampi (**d**; AUC increase from 107.9±21.6 to 173.3±24.8 ms; *n*=8; *P*=0.0012, paired *t*-test). Bars, mean; error bars, s.e.m.; circles, individual experiments.

Does the tonic current in ‘epileptic’ slices result from increased synaptic GABA release or from the reversal of GABA transport? In the latter case, inhibiting GATs should decrease the extrasynaptic GABA concentrations and thus reduce the tonic conductance in hippocampal neurons. In contrast, application of SKF89976A resulted in an outward shift in *I*_hold_ (Fig. 4a; 27.9±7.1 pA, *n*=6, *P*=0.011, paired *t*-test) indicating that inhibition of GAT-1 increases GABA_A_R-mediated conductance in hippocampal neurons under conditions that promote the occurrence of epileptiform discharges. The effect of SKF89976A on the *I*_hold_ is qualitatively similar to that in the normal Mg^2+^ aCSF (that is, in the absence of epileptiform activity; 15.7±3.4 pA, *n*=5, *P*=0.0102, paired *t*-test; Supplementary Fig. 3). This increase is likely to be due to the accumulation of synaptically released GABA. To test this, we preincubated slices in 1 μM concanamycin and performed recordings in the presence of tetrodotoxin, that is, in conditions with no vesicular GABA release. Indeed, GAT-1 inhibition in these experiments had little effect on the *I*_hold_, reflecting the lack of detectable GABA uptake in the absence of synaptic neurotransmission (Fig. 4b).

We also noted that the inhibition of glial transporter GAT-3 in slices generating epileptiform activity did not reduce tonic GABA_A_R conductances either. If anything, SNAP5114 produced a small, statistically insignificant outward shift in the *I*_hold_ (3.5±2.8 pA; *n*=5; *P*=0.22, paired *t*-test; Supplementary Fig. 4a), suggesting that GAT-1 activity remains the major factor that determines [GABA]_e_ detected by principal cells under epileptogenic conditions.

The lack of an inward shift in *I*_hold_ (that is, any decrease in GABA-mediated tonic current) measured between epileptiform bursts following GAT-1 or GAT-3 inhibition argues against a significant overall contribution of GAT-mediated GABA release to the accumulation of ambient GABA in ‘epileptic’ slices. GAT reversal, however, may still occur transiently, during pathological discharges. To test if this is the case, we compared the kinetics of field potential burst-associated large GABA_A_R transients with and without GABA transporters inhibition. It has been shown that, following concurrent activation of multiple release sites, for example, during evoked release, GABA uptake limits the activation of peri- and extrasynaptic GABA_A_Rs^36^. Thus, GAT blockade would be expected to prolong GABA_A_R-mediated currents during epileptic discharges if transporters operate in forward mode. SKF89976A considerably increased the duration of GABA_A_R-mediated currents (Fig. 4c), indicating that GAT-1 is actively engaged in clearing GABA from the extracellular space during burst discharges. This was evident not only in slices from control animals, but also in slices from chronically epileptic rats (Fig. 4d). The increase in the decay kinetics of the GABA_A_R-mediated transients was similar in both groups, suggesting that epileptogenesis is not associated with compensatory reversal of GAT-1 operation. In line with the limited contribution of GAT-3 to the level of ambient GABA detected by hippocampal pyramidal neurons in the presence of functional GAT-1 uptake, application of SNAP5114 did not alter the decay of large GABA_A_R transients (*n*=4, the area under GABA_A_R-mediated transients in SNAP5114 was 99.6±1.5% of control; *P*=0.54, paired *t*-test; Supplementary Fig. 4b).

Our *in vitro* and *in silico* experiments thus argue against the reversal of GAT-1 during epileptiform activity and suggest that increased vesicular GABA release during intensive firing of interneurons prevents GAT-1-mediated GABA efflux.

## Discussion

GABA transporters in neurons and astrocytes operate close to their equilibrium potential and therefore can reverse with depolarization potentially generating non-vesicular release of the neurotransmitter into the extracellular space^11^,^12^. Such observations have prompted the idea that transporter-mediated GABA efflux (reversal mode) may occur following K^+^ or glutamate-induced cell membrane depolarizations that accompany increased network activity and, therefore, can provide additional inhibition during epileptic discharges. However, this conjecture has remained speculative, mainly because electrophysiological experiments to support this have assessed GAT-1 reversal in the absence of synaptic activity^15^,^16^,^25^, which deviates from the scenario involving physiological or pathophysiological increases in brain network activity. Our previous work using *in vivo* microdialysis has demonstrated that concurrent inhibition of GABA transport and K^+^-induced neuronal depolarization induces a several-fold larger increase in [GABA]_e_ than does K^+^-induced depolarization alone, arguing against transporter-mediated efflux of GABA^17^. Here we examined this issue further by using detailed computational modelling and experimental recordings from acute hippocampal slices.

Using outside-out ‘sniffer’ patches to detect extracellular GABA *in vitro* and quantitative microdialysis *in vivo*, we previously found that the extracellular GABA concentration is maintained at a very low level and is likely to be less than 200 nM (ref. 22). This is in line with estimates of extracellular GABA in slices based on tonic GABA_A_R-mediated currents^37^, but somewhat lower than 0.8–2.4 μM suggested by early microdialysis studies^38^,^39^,^40^,^41^. However, the use of anesthesia during measurements and a short time between the probe implantation and sample collection in the latter studies may have overestimated [GABA]_e_. Conservative estimates of cytosolic GABA, Na^+^ and Cl^−^ concentrations used in the kinetic model here (see Methods) resulted in [GABA]_e_ close to that measured above: ~200 nM at an assumed resting membrane potential of −70 mV (for example, see refs 42, 43).

An important conclusion that follows from our simulations is that even in the absence of release, the reverse mode of GAT-1 operation in the hippocampus can occur only transiently, simply because the presence of newly released GABA will halt the reverse mode. In other words, on sustained depolarization, these transporters will only briefly work in reverse until [GABA]_e_ has reached the steady-state level for the new *V*_m_. Once this new level of extracellular GABA is reached, transporters will remain inactive (dormant). Under continuous uptake triggered by synaptic activity, the sustained subthreshold depolarization is unlikely to trigger even a transient reversal. Furthermore, GABA transporter reversal does not occur even when presynaptic terminals populated with GAT-1 transporters generate trains of action potentials. Although [GABA]_e_ in the presence of presynaptic spikes will be higher, this simply reflects the reduced GABA uptake during increased action potential-dependent GABA release. This sequence of events involving reduced GABA uptake suggests that high frequency interneuronal firing can enhance extrasynaptic spillover of GABA thus boosting activation of perisynaptic GABA_B_ receptors^44^.

Our modelling results indicate that in the presence of synaptic GABA release the reversal of GABA transport due to membrane depolarization is unlikely, mainly because synaptically released GABA contributes significantly to [GABA]_e_ (*in vivo* blocking action potential-dependent release decreases it by ~75% (ref. 17)). We also note that the use of *E*_GAT_ in determining the mode of GAT-1 operation is limited, because this parameter will dynamically change depending on the fluctuations of GABA, Na^+^ and Cl^−^, both inside and outside cells.

A number of clinical and animal studies of ictogenesis have shown that the increased excitatory drive may mask a massive recruitment of inhibitory neurons during both ictal and interictal events^45^,^46^,^47^,^48^. Indeed, here we demonstrate that epileptiform activity is accompanied by intense synaptic GABA release. Consistent with theoretical predictions, we did not observe a reduction in tonic GABA_A_R-mediated currents on blockade of neuronal GABA transporters. Inhibition of GAT-1 had qualitatively similar actions on picrotoxin-sensitive tonic currents under conditions designed to promote epileptiform activity and in quiescent conditions, suggesting that these transporters continue to operate in the forward mode despite hyperexcitable conditions and increased network activity. In contrast, the inhibition of GAT-1 in slices preincubated in concanamycin, and thus void of synaptic release, did not lead to increased tonic GABA_A_R currents. This argues that the enhanced synaptic GABA release, which accompanies epileptiform discharges in 0 Mg^2+^, compels the transporters to operate in the forward mode.

Astroglia have been suggested to be an important source of extracellular GABA in various brain regions, and reversed GABA transport^20^,^49^,^50^,^51^,^52^ as well as channel-mediated release^53^ have been implicated. In contrast to, for example, thalamus, where GAT-3 is abundantly expressed^33^,^54^, and in line with previous studies^17^,^33^, our results indicate that the GABA concentration in the hippocampus detected by pyramidal cells is primarily controlled by GAT-1. GAT-3 activity, on the other hand, in the presence of GAT-1-mediated uptake does not have a detectable impact on the tonic GABA_A_ receptor-mediated conductance in hippocampal pyramidal neurons. Although spatially segregated action of GAT-3 cannot be excluded^5^, and our data do not allow us to unequivocally rule out GAT-3 reversal during epileptic discharges^18^,^19^, reversed uptake by GAT-3 would be expected to further facilitate the forward mode of GAT-1 operation.

An alternative mechanism for GAT-1 reversal may involve the collapse of Na^+^ gradient, which drives the transporters in the forward mode, and a gradual accumulation of Cl^−^ following extensive seizure activity, for example, see refs 29, 30. However, such ionic changes will in the first place reduce the efficacy of the GABA uptake; and our simulations show that in the presence of synaptic release the reversal of GAT-1 in this case is still unlikely. One prediction from these simulations is that impaired Cl^−^ extrusion in chronic epilepsy^55^ may result in an increased [GABA]_e_, which in turn could partly explain why tonic currents are not only maintained, but often are increased in epileptic animals^56^,^57^. Similarly, in the neonatal brain, [GABA]_e_ may be maintained at higher levels due to developmental differences in the Cl^−^ homeostasis^55^.

Furthermore, our results show an increased GABAergic synaptic drive onto CA1 pyramidal neurons in 0 Mg^2+^ perfusion solution, providing indirect evidence that Na^+^ accumulation in interneurons is limited, as it would be expected to manifest itself in the abortion of action potential-dependent synaptic GABA release. Indeed, interneurons, especially fast spiking cells, display a higher Na^+^/K^+^-ATPase activity than that of pyramidal neurons, arguably to facilitate high frequency firing^58^. Thus, although GAT reversal due to [Na^+^]_in_ accumulation is plausible under extreme pathological conditions (when neuronal Na^+^/K^+^-ATPase fails to maintain low intracellular Na^+^ concentrations^59^, or in the rare circumstance when GAT-1 and GABA are expressed by excitatory neurons^60^), physiologically relevant network activity is unlikely to be associated with such a failure.

Several recent studies have proposed that certain types of interneurons may cease firing during epileptic discharges; however, the transition of interneurons into depolarization block is usually preceded by a period of their hyperactivity^61^,^62^. Recordings from patients with epilepsy, as well as animal studies, have also demonstrated early recruitment of interneurons before seizure precipitation^46^,^48^ or at the beginning of interictal discharges^62^. In this scenario (Fig. 1e), ambient GABA elevation due to intense synaptic release before the reduction in GABAergic neurotransmission will still be expected to counteract the reversal of GATs. However, it is worth noting that many interneurons do not display depolarization block, and in our experience even those slices that generate prolonged ictal-like activity display a sustained increase of the GABAergic drive onto pyramidal cells. Furthermore, in the present study, we did not observe GAT-1 reversal during pathological discharges in the hippocampus from chronically epileptic animals. Somewhat counterintuitively, our simulations suggest that reducing the number of GABAergic synapses due to the loss of interneurons commonly observed in epilepsy, for example, refs 63, 64, should make GAT-1 reversal even less likely by decreasing the ratio of volume fraction occupied by GABAergic terminals and the extracellular space.

In conclusion, our results argue that, with synaptic GABAergic neurotransmission intact, GAT-1 operation in the reverse mode is unlikely to occur even during extreme neuronal activity. When GABAergic transmission fails, the transporter could reverse but only transiently. Thus, extracellular GABA concentrations in the brain are set, depending on the level of network activity, by synaptic GABA release and the rate of GABA uptake, rather than by GAT-1-mediated efflux.

## Methods

Either 3–4-week-old or adult male Sprague–Dawley rats (Harlan Laboratories Inc, Bicester, UK) were used in the experiments. Animals were kept under standard housing conditions with a 12-h light–dark cycle and free access to food pellets and drinking water. Animal procedures were subject to local ethical approval and followed the UK Home Office Animal (Scientific Procedures) Act, 1986.

### *In vitro* electrophysiology

*In vitro* electrophysiological recordings were performed in acute hippocampal slices prepared from 3–4-week-old control rats and adult chronically epileptic and sham-control animals. After decapitation, brains were rapidly removed and hippocampi were dissected. Transverse hippocampal slices (350-μm thick) were cut with a Leica VT1200S vibratome (Germany) in an ice-cold sucrose-based solution containing (in mM): sucrose (70), NaCl (80), KCl (2.5), MgCl_2_ (7), CaCl_2_ (0.5), NaHCO_3_ (25), NaH_2_PO_4_ (1.25) glucose (22), bubbled continuously with 95% O_2_+5% CO_2_ to yield a pH of 7.4. Slices were allowed to recover in a sucrose-free aCSF solution (in mM): NaCl (119), KCl (2.5), MgSO_4_ (1.3), CaCl_2_ (2.5), NaHCO_3_ (26.2), NaH_2_PO_4_ (1), glucose (22), bubbled with 95% O_2_ and 5% CO_2_ in an interface chamber for at least 1 h at room temperature before being transferred to a submerged recording chamber. Modified aCSF (nominally 0 mM Mg^2+^ and 5 mM K^+^) was used to induce epileptiform activity. To facilitate rapid generation of epileptiform discharges slices were perfused with the solution on both sides. All recordings were done at 32 °C. GABA_B_ receptors were blocked in all experiments by 1 μM CGP55845.

Field potential recordings from stratum pyramidale were performed with 1–2 MΩ glass electrodes field with aCSF. Visualized whole-cell voltage-clamp recordings were performed from CA1 pyramidal neurons using infrared differential interference contrast imaging system. Recording pipettes (3–5 MΩ) were filled either with a high Cl^−^ internal solution containing (in mM): CsCl (120), HEPES (10), EGTA (2), NaCl (8), MgCl_2_ (0.2), Mg-ATP (2), Na-GTP (0.3), QX-314 Br^−^ salt (5), pH 7.2, 290 mOsm (experiments in Fig. 2, neurons voltage clamped at −70 mV), or with a low Cl^−^ internal solution, containing (in mM): Cs-methanesulfonate (120), HEPES (10), EGTA (0.2), NaCl (8), MgCl_2_ (0.2), Mg-ATP (2), Na-GTP (0.3), QX-314 Br^−^ salt (5), pH 7.2, 290 mOsm (all other whole-cell recordings; neurons held at 0 mV). Recordings from cells voltage clamped at −70 mV were performed in the presence of AMPA/kainate and NMDA receptor blockers NBQX (25 μM) and D-AP5 (50 μM) respectively. Na^+^-channel blocker tetrodotoxin (1 μM) was also included in the perfusion solution. In the absence of glutamate receptor blockers (when epileptiform activity was induced), GABA_A_R-mediated currents were recorded from neurons voltage clamped at 0 mV (close to the reversal potential of glutamatergic currents) using low Cl^−^ internal solution. To prevent the contribution of NMDA receptors MK801 (1 mM) was included in the recording pipettes in these experiments. Tonic GABA_A_R-mediated currents were measured as changes in holding current 10 min following application of GAT inhibitors or after adding GABA_A_R antagonist picrotoxin (100 μM). To avoid bias due to increased frequency of synaptic currents in recordings from slices with epileptiform activity, the method of Gaussian fit was used to determine the mean holding current^4^. To establish the effect of GAT inhibitors on the decay of burst-associated GABA_A_R-mediated transients, recurrent events were identified using the threshold-detection protocol in Clampfit 10.0 set at least twice exceeding the size of the background activity. At least 20 events were averaged for each condition.

Series resistance (*R*_s_) was monitored throughout the experiment using a –5-mV step command. Cells showing a >20% change in *R*_s_, or values >25 MΩ, or an unstable holding current, were rejected. Recordings were obtained using a MultiClamp 700B amplifier (Axon Instruments, Foster City, CA, USA), filtered at 4 kHz and digitized at 10 kHz. Data acquisition and off-line analysis were performed using WinEDR 3.0.1 (University of Strathclyde, Glasgow, UK) and Clampfit 10.0 (Molecular Devices Corporation, USA) software.

GABA transporter inhibitors, tetrodotoxin, glutamate and GABA receptor blockers were purchased from Tocris Cookson (Bristol, UK) and Ascent Scientific (North Somerset, UK).

Two-tailed paired and unpaired Student’s *t*-test was used for statistical analysis of electrophysiological data. Differences were considered significant when *P*<0.05. A Holm–Bonferroni correction for multiple comparisons was used to adjust the level of significance for data presented in Fig. 2. Data are shown as mean±s.e.m.

### Perforant path (PP) stimulation epilepsy model

Chronic epilepsy was induced in male Sprague–Dawley rats (290–350 *g*) rats as described previously^65^. Rats were operated under isoflurane anesthesia to implant a bipolar stimulating electrode and a unipolar recording electrode into the right angular bundle (8.1 mm anterior and 4.4 mm lateral to bregma) and dentate gyrus (−4.4 mm anterior and −2.5 lateral to bregma), respectively. A silver earth electrode was positioned subcutaneously and electrodes were mounted into a six-channel plastic pedestal and secured using skull screws and dental cement (Kemdent, UK). To ensure the correct location of the electrodes, stimulation of the PP was performed while monitoring field potential responses from the dentate gyrus. After at least 7 days of recovery status epilepticus (SE) was induced by 50 μs pulses continuously applied to the PP at 20 Hz for 2 h. Animals displaying no seizures were excluded from experiments. SE was allowed to continue in the absence of stimulation for 3 h and was then terminated by intraperitoneal administration of diazepam (<20 mg kg^−1^). Animals were then rehydrated with saline (0.9% w/v, subcutaneously). Chronically epileptic rats were used for *in vitro* electrophysiology 4–5 weeks after SE induction. Sham-control animals underwent similar surgery procedures without SE induction.

### Modelling

Steady-state and pre-steady-state GAT-1 transmembrane currents were simulated using a previously published kinetic scheme^21^. All parameters of the state transitions were the same as in the original publication. Pascal 7.0 code was used for numerical integration of the set of differential equations. Steady-state values were calculated using the Gauss–Seidel method (also known as the Liebmann method). Computer code used for simulations is available on request.

[GABA]_e_ dynamics were calculated in the volume of 1 × 10^3^ μm^3^ using PASCAL model according to the following equation:




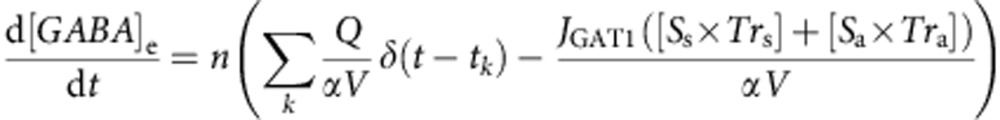




where *δ* is a delta function.

Simulated extracellular space was populated by *n* uniformly distributed synapses. Each synapse independently released *Q*=3,000 GABA molecules^66^ at the time *t*_*k*_*=f*^−1^, where *f* is the frequency of synaptic release. Each terminal was considered as a half-sphere with a radius of 0.25 μm, and was connected to a 22-μm-long segment of an axon. Synaptic surface, *S*_s_, was assumed to have *Tr*_s_=3,000 GAT-1 molecules μm^−2^ resulting in ~1,200 GATs per bouton; axon segments had *Tr*_a_=640 transporters per μm length, *S*_a_ (ref. 24). The density of GABAergic terminals was chosen to match experimentally observed values^23^. We used volume fraction of extracellular space *α*=0.14 similar to the values observed in the CA1 region of the hippocampus^26^,^27^. Following each release event, GABA molecules instantly filled simulated extracellular space uniformly increasing GABA concentration. The model made no assumptions regarding the dynamics of cytosolic–vesicular GABA exchange, and to preserve the overall GABA content any increase in the extracellular GABA was accompanied by a corresponding decrease in the cytosolic GABA. The rate of GABA concentration change depended on the current generated by *m*=([*S*_s_ × *Tr*_s_]+[*S*_a_ × *Tr*_a_]) molecules of GAT-1, *mJ*_GAT1_. Depending on the direction of the current, [GABA]_e_ either increased or decreased. It was assumed that a single charge transfer by the transporter corresponds to the translocation of one GABA molecule. Computations were carried out using a 64-core in-house computer cluster^67^ with software environment optimized in collaboration with Sitrus LLC (Boston).

Voltage changes due to presynaptic spiking were simulated as step-like jumps of 120 mV in amplitude each having a duration of 2 ms at the frequency of *f*_AP_. Release probability of GABAergic synapses was assumed to be 0.5 (ref. 32), so the frequency of release was twice lower than the frequency of spiking.

Internal concentrations of [Na^+^]_in_=7 mM and [Cl^−^]_in_=7 mM were chosen to fall within physiologically relevant range of 5–10 mM estimated for neurons, for example, refs 29, 68, 69, 70; external concentrations of these ions were as in the aCSF used in the experiments ([Na^+^]_e_=146.2 mM, [Cl^−^]_e_=126.5 mM). In some simulations (Supplementary Fig. 2), [Cl^−^]_in_ was increased to 30 mM (to mimic proposed seizure-induced Cl^−^ accumulation^29^,^30^) and [Na^+^]_in_ to 15 mM (to account for the spike-associated influx^31^; presynaptic terminals may fail to sustain action potential generation at higher values in the Hodgkin–Huxley model). Cytosolic GABA concentration in cortical neurons remains unknown and values around 2 mM are considered to be justified^15^. We have considered [GABA]_cyt_ as a dynamically changing concentration calculated as follows:




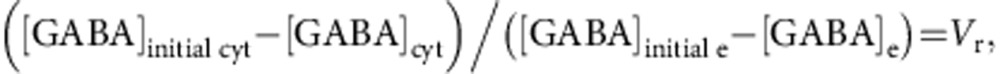




where *V*_r_~42 is the ratio between the extracellular and intracellular space. The initial cytosolic GABA concentration, [GABA]_initial cyt_, was set at 2 mM. Assuming [GABA]_e_=100 nM (ref. 22), this resulted in *E*_GAT_=−74.25 mV (close to the resting membrane potential of −70 mV used in our simulations). We have also tested GAT-1 kinetics with intracellular GABA ranging between 1 and 4 mM. Altering cytosolic GABA concentration did not change the shape of the current–voltage relationship of GAT-1 and *E*_GAT_ sensitivity to extracellular GABA (Supplementary Fig. 5a,b). The results of our simulations suggest that, for a given subthreshold, *V*_m_ (for example, −70 mV), elevating [GABA]_cyt_ will result in an increased basal extracellular GABA level (that is, the equilibrium achieved in the absence of synaptic release Supplementary Fig. 5c).

## Additional information

**How to cite this article:** Savtchenko, L. *et al*. Synaptic GABA release prevents GABA transporter type-1 reversal during excessive network activity. *Nat. Commun.* 6:6597 doi: 10.1038/ncomms7597 (2015).

## Supplementary Material

Supplementary Information Supplementary Figures 1-5
