# Supplementary material for: Synaptic GABA release prevents GABA transporter type-1 reversal during excessive network activity
Source: Nat Commun. 2015 Mar 23;6:6597. doi: 10.1038/ncomms7597 (PMC4374149; doi:10.1038/ncomms7597)
Supplement: Supplementary Information — Supplementary Figures 1-5 [file ncomms7597-s1.pdf]

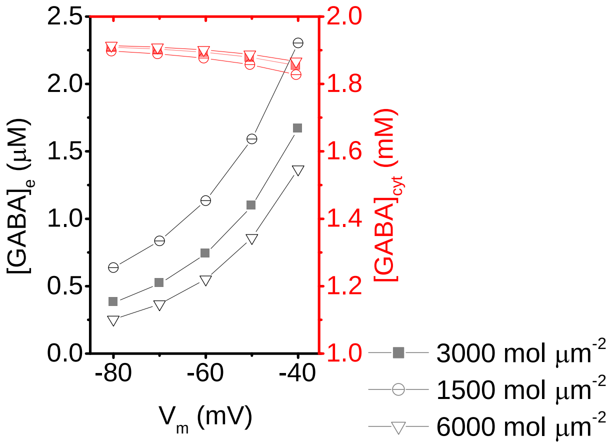

Supplementary Figure 1. **GABA concentrations at different GAT-1 densities.** Computer simulations showing membrane potential dependence of the steady-state extracellular GABA concentrations ($[GABA]_e$; *black*) and cytosolic GABA concentrations ($[GABA]_{cyt}$; *red*) at different GAT-1 densities. Synaptic release rate is 2 Hz; filled symbols show corresponding values from Fig. 1d for comparison.

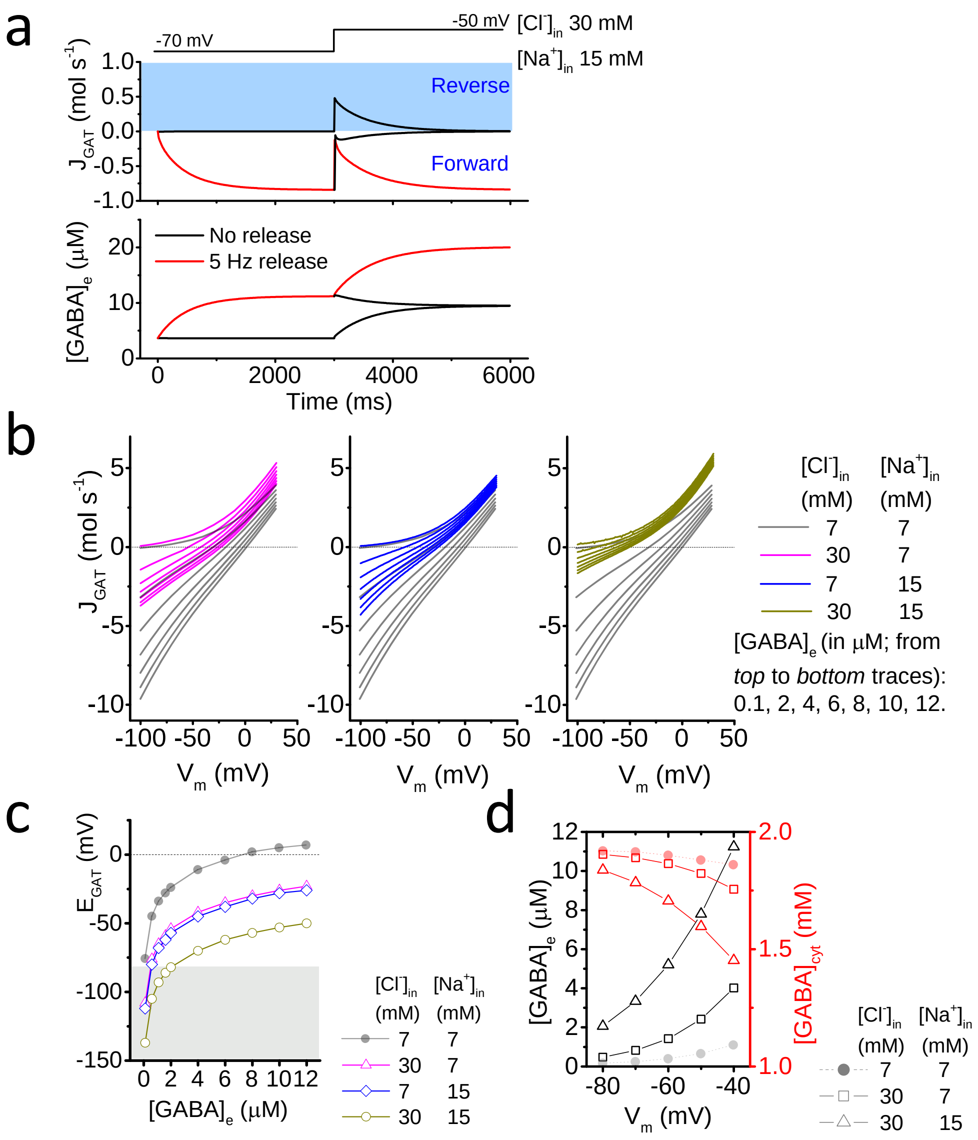

Supplementary Figure 2. GAT-1 activity and GABA concentrations at high levels of internal Cl^- and Na^+. (**a**) Computer simulations showing the effect of depolarization on the dynamics of GAT-1 operation and extracellular GABA concentration ($[GABA]_e$) with (*red*) and without (*black*) synaptic GABA release. (**b**) Current-voltage relationships of the steady-state GAT-1-mediated current (J_{GAT}, molecules per s; negative values – forward mode, positive values – reverse mode) at different concentrations of extracellular GABA ($[GABA]_e$ as in Fig. 1a). A family of traces from Fig. 1a ($[Cl^-]_{in}$ = 7 mM; $[Na^+]_{in}$ = 7 mM) is shown in *gray* for comparison. (**c**) Dependence of GAT-1 reversal potential (E_{GAT}) on the concentration of extracellular GABA at different values of internal Cl^- and Na^+. *White area*, physiologically relevant membrane potentials. (**d**) Membrane potential (V_m) dependence of the steady-state cytosolic ($[GABA]_{cyt}$) and extracellular GABA at different values of internal Cl^- and Na^+ in the absence of synaptic release (filled symbols show corresponding values from Fig. 1c for comparison).

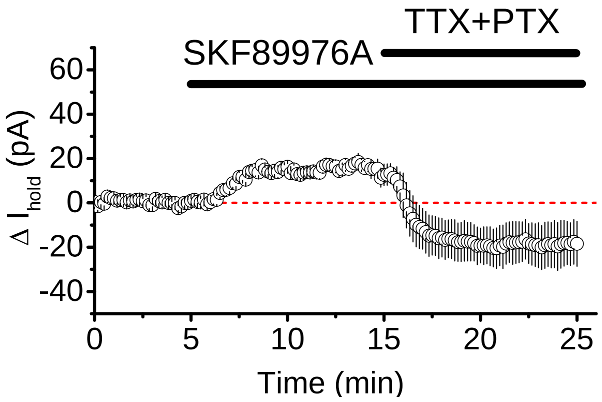

Supplementary Figure 3. **Inhibition of GAT-1 increases tonic GABA$_A$R-mediated currents.** Holding current (I_{hold}) changes in CA1 pyramidal neurons induced by application of SKF899976A in the absence of epileptiform activity (normal Mg^{2+} aCSF; V_{hold} = 0 mV; low Cl^- intracellular recording solution; c.f. Fig. 4a; n = 5). TTX, tetrodotoxin; PTX, picrotoxin. Error bars, s.e.m.

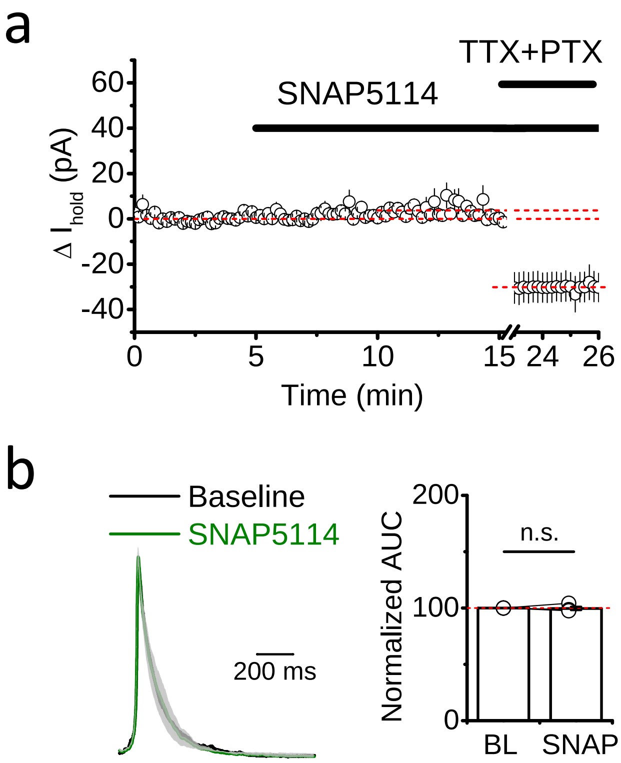

Supplementary Figure 4. GAT-3 impact on GABA$_A$R currents during epileptiform activity. (**a**) Tonic GABA$_A$ receptor-mediated currents in CA1 pyramidal neurons following application of SNAP5114 (100 µM) during ongoing epileptiform activity (n = 5). (**b**) Mean normalized traces of GABA$_A$R transients (*gray*: s.e.m.) show lack of effect of GAT-3 inhibition on the kinetics of burst-associated GABA$_A$R transients in pyramidal neurons (area under the curve, AUC, baseline: 60.9 ± 14.8 ms, in SNAP5114: 60.3 ± 14.1 ms; n = 4; p = 0.54, paired t-test). TTX, tetrodotoxin; PTX, picrotoxin. Bars, mean; error bars, s.e.m.; circles, individual experiments.

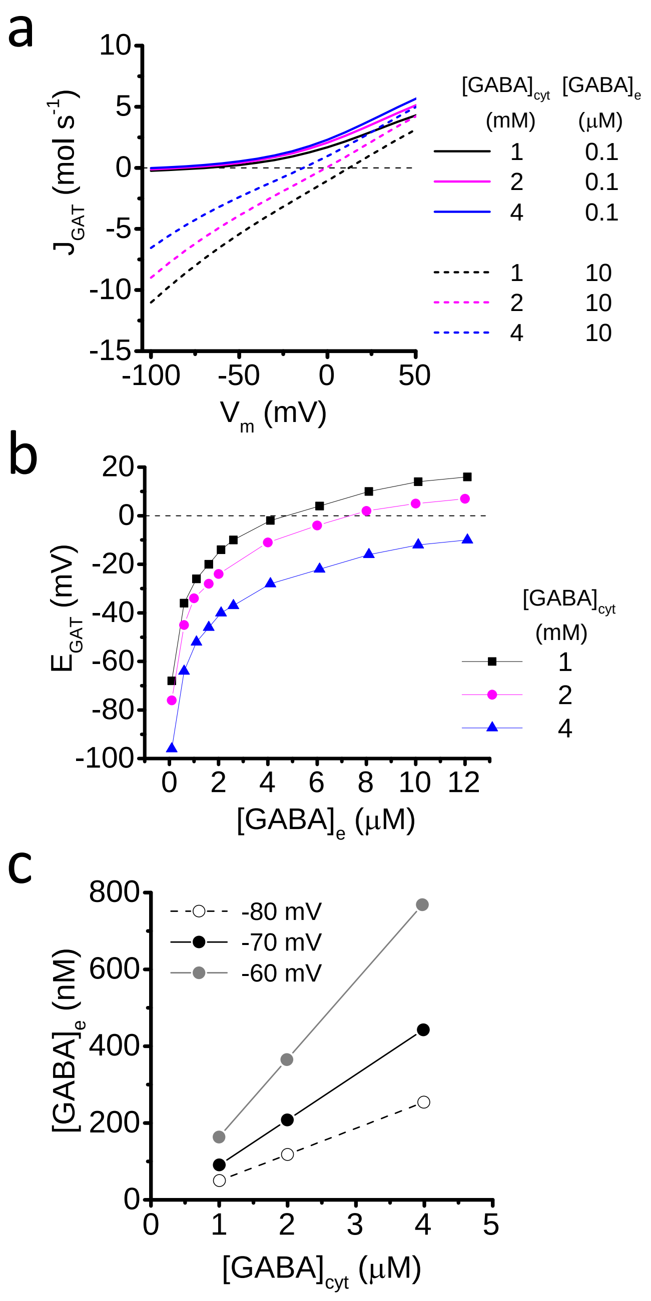

Supplementary Figure 5. **GAT-1 simulations at different cytosolic GABA concentrations.** (**a**) Current-voltage relationship of the steady-state GAT-1-mediated current (J_{GAT}, molecules per s; negative values – forward mode, positive values – reverse mode) at different concentrations of the cytosolic ($[GABA]_{cyt}$) and extracellular ($[GABA]_e$) GABA. (**b**) Dependence of GAT-1 reversal potential (E_{GAT}) on the concentration of extracellular GABA. (**c**) Steady-state extracellular GABA concentrations vs. cytosolic GABA at different subthreshold membrane potentials.
